# Practice visitations in primary care to improve performance of cardiovascular risk management: an observational study

**DOI:** 10.3399/BJGPO.2023.0213

**Published:** 2024-07-10

**Authors:** Geert HJM Smits, Michiel L Bots, Monika Hollander, Ardine de Wit, Sander van Doorn

**Affiliations:** 1 Julius Center for Health Sciences and Primary Care, University Medical Center Utrecht, Utrecht University, Utrecht, Netherlands; 2 Vrije Universiteit Amsterdam, Amsterdam, Netherlands

**Keywords:** practice visitations, cardiovascular diseases, cardiovascular risk management, primary health care, prevention, observational study

## Abstract

**Background:**

Despite programmatic protocolised care and structured support, considerable variation is observed in completeness of registration and achieving targets of cardiovascular risk management (CVRM) between individual GPs in the Netherlands.

**Aim:**

To determine whether completeness of registration and achieved targets of cardiovascular risk factors improves with practice visitation.

**Design & setting:**

Observational study utilising the care group's database (2016–2019), comparing changes in registration and achieved targets in non-visited practices and visited practices.

**Method:**

We compared completeness scores of registration and scores of targets achieved before visitation and 1 year after visitation. Data were analysed on patient level and GP level. Separate analyses were performed among GPs who were ranked in the lower 25% of score distributions.

**Results:**

We observed no clinically relevant improvements in completeness of registration and targets achieved in 2017, 2018, and 2019 that could be attributed to visitations in the previous year, both on individual patient level and on aggregated level per general practice. In practices ranked in the lower 25% of the distribution, improvements over time were clinically relevant and larger than the overall changes. Yet, these findings were irrespective of the number of practice visitations.

**Conclusion:**

Practice visitations in our setting did not seem to lead to improvements in practice performance, nor in completeness of registration of risk factors or in reaching predefined target goals for cardiovascular risk factors.

## How this fits in

Although comprehensive care group support was identical for all affiliated general practices from the start with integrated cardiovascular risk management (CVRM) care in 2010, considerable variation in performance between practices regarding completeness of registration and reaching predefined targets was observed after a few years. To our knowledge, individual practice visitations with the aim of reducing interpractice variation, have not previously described. Low-and-moderate performing practices were visited two or three times a year, while average and good-performing practices were not visited or were visited once a year. Completeness of registration and reaching targets did not seem to improve, regardless the number of visitations.

## Introduction

Since the introduction of national and international guidelines, cardiovascular risk management (CVRM) is increasingly organised and implemented by primary care groups in the Netherlands.^1,2^ Primary care groups are responsible for the provision of a high quality, evidence-based CVRM care programme for affiliated practices. The implementation of the CVRM care programme was delegated to a practice nurse (PN) supervised by the GP. The PN guided eligible patients with medication adjustments and supported with stopping smoking, changing unhealthy food habits, and increasing exercise. The care group supported practices with protocols, small group interactive education, audit and feedback (A&F), outreach visits, and reminders, as it is known that a comprehensive approach is most beneficial to improve implementation.^3^ A&F is a widely used component in clinical programmes, but its effect is shown to be modest.^4–8^ To what extend A&F is of value to programmatic CVRM is not yet known. Therefore, the aim of the present study was to investigate whether A&F delivered during practice visitations leads to improvements in completeness of registration and reaching predefined targets among participating practices in the CVRM care programme between 2016 and 2019.

## Method

### Study design and study population

We carried out a dynamic cohort study using data that were routinely collected from 128 practices affiliated to the PoZoB primary care group in 2016. The care group implemented a nurse-led integrated CVRM care programme between 2010 and 2013. The practices were located in and around Eindhoven, in the south-east of the Netherlands. They were a mixture of rural, semi-rural, and urban practices and can be considered representative for the Dutch context.

### Data collection

In 2016 data were collected from 48 258 patients eligible for integrated CVRM care. Conditions for eligibility for the CVRM care programme were based on the National CVRM guideline 2012 and have been described in detail elsewhere.^9,10^ Patient data were collected in the care group's multidisciplinary registration (Care2U: C2U). For the present analyses, we excluded practices that changed hands between 2016 and 2019, because starting GPs often have other priorities than organising disease management programmes. We therefore used data from 128 practices (40 525 eligible patients) where the same GP was employed between 2016 and 2019.

### Care group support and benchmark indicators

Between 2010 and 2015, the care group supported with work protocols, peer group meetings for the PN, and education for GPs and PNs on cardiovascular-related topics. From 2016 onwards, the care group started publishing quarterly benchmark reports in which individual practices could compare their performance with overall care group performance. Indicators used for defining performance were (i) registration of: systolic blood pressure (SBP), low-density lipoprotein (LDL)-cholesterol, estimated glomerular filtration rate (eGFR), body mass index (BMI), range of exercise (based on the Dutch norm for healthy physical exercise), alcohol intake and smoking status; and (ii) outcomes: the proportion of patients on target for SBP (≤140 mm Hg), LDL-cholesterol (≤2.5 mmol/l), BMI (≤25 kg/m^2^), the proportion of non-smoking patients, the proportion on blood pressure-lowering treatment and the proportion on lipid-modifying treatment. For every indicator the care group mean and standard deviation (SD) was calculated, which was used as a benchmark for individual practices. Mean plus one SD was defined as 'best practice' and mean minus two SDs as 'minimal norm'. Standards were discussed in staff meetings and confirmed in the care group Advisory Board of General Practitioners. With these standards set, every individual practice was given an impression about their performance with the overall care group as comparator.

### Practice visitations

In 2016 the care group started with practice visitations to discuss performance based on the benchmark data and offer support to improve the organisation of CVRM. Important goals of the visitations were building a relationship of trust and emphasising the partnership between practice and care group. In case of low performance, the care group supported with the analysis of possible causes, made practices primarily responsible and co-owner of the solution, and provided temporary guidance. Visitations were performed by a university-trained staff member with an additional education in management and organisation. Practices having difficulties with organising and implementing the CVRM care programme (discontinuity of staff, insufficient hours for the PN, no regular consultations between GP and PN) and practices with a number of indicators below the minimal norm were initially prioritised for visitation. Practices could also request a visitation. The visitations usually lasted 1–1.5 hours and in almost all cases were carried out by one staff member. In exceptional cases the visit was carried out by two persons if a specific problem needed to be explained. Practice members attending the visits were the GP, the PN, and in some cases the manager of a health centre. Based on a standard 8-item questionnaire shown in Box 1, practice organisation and performance was discussed with the GP and the PN. An example of items discussed during a practice visitation is given in Supplementary Box 1.

Box 1 **Items discussed with the practice during visitation**

- Is the practice using a result-oriented approach (focus on how to interpret information from the quarterly reports and improving to at least the minimum standard)?
- Is the practice applying or, if necessary, deviating from guidelines?
- Is there regular and structured consultation between GP and practice nurse (PN) and is there agreement on the follow-up policy so the PN feels supported by the GP?
- Is there adequate mail processing and registration in the multidisciplinary information system?
- Does the PN have sufficient hours in relation to the workload?
- Is there discontinuity owing to illness of the GP or the PN?
- Are there major differences in practice population?
- Are there linking problems with a laboratory, electronic registration system, or multidisciplinary registration system?

### Data analyses

We started with the registration of SBP, LDL-cholesterol, eGFR, BMI, alcohol intake, (self-reported) range of exercise, and smoking status in 2016 on an individual patient level. An item registered = 1, not registered = 0, adding up to an individual patient score between 0 and 7. This was followed by the same procedure for reaching the predefined targets: SBP ≤140 mm Hg, LDL-cholesterol ≤2.5 mmol/l, BMI ≤25 kg/m2, not smoking, use of blood pressure-lowering medication and the use of lipid-modifying medication, adding up to an individual patient score between 0 and 6. Next, the mean number of scored items per individual patient in practices with no visit (V0), with one visit (V1), and with more than one visit (V2/3) in 2016 were compared with the scores observed in those patients in 2017 (the next year). As the study was designed as a dynamic cohort study, patients could leave the study and new eligible patients could enter the study. We compared only those patients who were in the study for both time periods. A similar procedure was applied for the 2017–2018 and 2018–2019 comparisons. Descriptive analyses were run and presented for overall and in strata of visitation frequency (mean with SDs), crude difference in completeness, and reaching targets (mean and standard error). In order to evaluate whether the observed differences were confounded, multivariable linear regression models were run in which age, sex, care programme (eligible for secondary prevention or eligible for primary prevention), and GP were taken into account as potential confounders. As sensitivity analyses, we repeated the analyses with aggregated scores on GP level, where now visitations involved the practice rather than individual patients.

### Patient involvement

Since the study was aiming at data derived from the multidisciplinary information system for integrated care, patients were not actively involved.

### Ethical considerations

Data used for the analysis were pseudonymised when extracted from the multidisciplinary information system. Before uploading to the secure network, the data were encrypted, meaning that individual patient data were not identifiable during analyses.

## Results

The number of patients potentially reached through these visits is presented in Table 1. General cardiovascular characteristics of the population is presented in Table 2.

**Table 1.** Characteristics of the study population: practices, visitation, and number of patients potentially reached by the visitations

Year	2016	2017	2018
Practices participating in the study	128	128	128
Visitations per practice, *n*			
No visitation	38	37	27
1 visitation	87	71	79
2 visitations	3	16	20
3 visitations	0	4	2
Patients potentially affected through the visitation, *n*			
No visitation	11 492	11 814	8543
One visitation	26 678	20 999	23 508
Two or more visitations	1277	5642	7083
Total patients involved	39 447	38 455	39 134

**Table 2.** Mean characteristics of the studied population, by year

	2016 (*n* = 39 447)		2017 (*n* = 38 455)		2018 (*n* = 39 134)	
Mean age, years (SD)	69	10	69	10	70	11
Women, %	52		52		52	
High-risk patients without CVD, %	59		56		55	
Smoking, %	13		12		12	
Sufficient PA, %	76		76		76	
BPL-medication, %	63		66		68	
LM-medication, %	49		55		54	
eGFR ≥60 ml/min/1.73m^2^, %	79.8		78.6		79.7	
	**Mean**	**SD**	**Mean**	**SD**	**Mean**	**SD**
Alc, drinks/day	0.84	1.2	0.82	1.1	0.83	1.1
BMI, kg/m^2^	27.3	4.3	27.4	4.6	27.3	4.5
SBP, mmHg	136	15.7	136	15.5	135	15.5
LDL, mmol/l	2.8	0.91	2.7	0.89	2.4	0.82

Alc = alcohol. BMI = body mass index. BPL = blood pressure-lowering medication. eGFR = estimated glomerular filtration rate. LM = lipid-modifying medication. PA = physical activity, based on the Dutch norm for healthy movement. SBP = systolic blood pressure

### Completeness of registration

The mean score on completeness of registration using the individual patient data was 6.28 (SD 1.35) in 2016, 6.12 (1.71) in 2017, and 6.37 (1.33) in 2018 (Table 3). Results using individual patient data showed no clinically relevant improvements in completeness of registration overall. There was a tendency that improvements were larger in practices with two or more visitations compared with those with no or one visitation. Yet, the improvement in total score remained small in magnitude, despite reaching statistical significance also when confounding factors were taken into account (Table 3, last column). The mean scores using data aggregated on GP level were 6.00 (0.69) in 2016, 6.20 (0.83) in 2017, and 6.38 (0.26) in 2018 (Supplementary Table S1). Results using the data aggregated on GP level, followed the same pattern: no clinical relevant improvements overall and in practices with no visitations and one visitation, and small improvements in practices with two or more visitations in 2016 and 2017. Visitations in 2018 did not change completeness of registration overall, nor across strata of visitation on GP level in 2019, and analysis did not show different results in either direction, magnitude, or clinical relevance (Supplementary Table S1).

**Table 3.** Completeness of registration of CVRM information using individual patient records

	Mean completeness score (SD) year 1	Mean completeness score (SD) year 2	Mean difference between score (SE)(year 2 year 1)	Multivariable^a^ difference [99% CI]
**2016-2017**				
Overall	6.28 (1.35)	6.28 (1.39)	0.003 (0.010)	NA
0 visitations	6.33 (1.32)	6.28 (1.37)	–0.045 (0.018)	ref value
1 visitation	6.27 (1.35)	6.28 (1.39)	0.009 (0.011)	0.077 [0.021 to 0.133]
2 or more visitations	5.93 (1.55)	6.27 (1.41)	0.338 (0.058)	0.355 [0.208 to 0.503]
**2017-2018**				
Overall	6.12 (1.71)	6.37 (1.33)	0.250 (0.011)	NA
0 visitations	6.20 (1.53)	6.41 (1.29)	0.202 (0.018)	ref value
1 visitation	6.30 (1.34)	6.36 (1.34)	0.054 (0.013)	–0.089 [–0.14 to –0.032]
2 or more visitations	5.21 (2.68)	6.31 (1.39)	1.104 (0.041)	–0.152 [–0.239 to –0.065]
**2018-2019**				
Overall	6.37 (1.33)	6.34 (1.35)	–0.032 (0.009)	NA
0 visitations	6.38 (1.32)	6.26 (1.43)	–0.120 (0.020)	ref value
1 visitation	6.35 (1.35)	6.34 (1.35)	–0.012 (0.012)	0.106 [0.044 to 0.167]
2 or more visitations	6.42 (1.27)	6.43 (1.25)	0.011 (0.021)	0.121 [0.041 to 0.201]

^a^Adjusted for year specific age, sex, care programme, and GP using a multivariable linear regression model with the difference in score as dependent variable. CI = confidence interval. CVRM = cardiovascular risk management. SD = standard deviation. SE = standard error

### Reaching predefined targets

The mean score on reaching the targets using the individual patient data was 3.21 (SD 1.30) in 2016, 3.28 (1.37) in 2017, and 3.51 (1.28) in 2018 (Table 4). Results based on individual patients showed no clinically relevant improvements in reaching targets overall nor in practices with no visitation and one visitation, and small improvements in practices with two or more visitations in 2016 and 2017. Visitations in 2018 did not show improvements in reaching targets overall in 2019, nor in strata across visitation. Adjustment for potential confounding factors did not affect the findings (Table 4, last column).

**Table 4.** Reaching predefined targets using individual patient records

	Mean number on target (SD) year 1^a^	Mean number on target (SD) year 2^a^	Mean difference between score (SE)	Multivariable^b^ difference [99% CI]
**2016-2017**				
Overall	3.21 (1.30)	3.36 (1.28)	0.144 (0.009)	NA
0 visitations	3.22 (1.31)	3.31 (1.28)	0.089 (0.017)	Ref value
1 visitation	3.22 (1.29)	3.37 (1.28)	0.147 (0.011)	0.067 [0.16 to 0.12]
2 or more visitations	3.02 (1.34)	3.59 (1.23)	0.573 (0.050)	0.450 [0.32 to 0.59]
**2017-2018**				
Overall	3.28 (1.37)	3.51 (1.27)	0.230 (0.010)	NA
0 visitations	3.32 (1.32)	3.53 (1.26)	0.215 (0.017)	Ref value
1 visitation	3.41 (1.25)	3.50 (1.28)	0.090 (0.012)	–0.091 [–0.144 to –0.038]
2 or more visitations	2.72 (1.72)	3.53 (1.25)	0.805 (0.030)	0.017 [–0.063 to 0.097]
**2018-2019**				
Overall	3.51 (1.28)	3.48 (1.26)	–0.0308 (-0.009)	NA
0 visitations	3.52 (1.29)	3.46 (1.26)	–0.0552 (0.012)	Ref value
1 visitation	3.52 (1.27)	3.48 (1.26)	–0.0416 (0.012)	0.060 [–0.052 to 0.065]
2 or more visitations	3.46 (1.28)	3.50 (1.24)	0.0344 (0.022)	0.132 [0.056 to 0.208]

^a^Targets are assessed for only those that have at least one measurement that determine the target score. ^b^Adjusted for year specific age, sex, care programme, and general practitioner using a multivariable linear regression model with the difference in score as dependent variable.CI = confidence interval. SD = standard deviation. SE = standard error.

The mean score on reaching targets using data aggregated on GP level was 3.08 (0.57) in 2016, 3.36 (0.64) in 2017, and 3.56 (0.46) in 2018. Results using the data aggregated on GP level showed no clinically relevant improvements overall and in practices with no visitation and one visitation, and small improvements in practices with two or more visitations in 2016 and 2017. Visitation in 2018 did not show improvements in reaching targets overall in 2019, nor across strata of visitation and analysis did not show different results in either direction, magnitude, or clinical relevance (Supplementary Table S2).

### Results among those GPs ranking lowest 25% of the distribution

From a clinical perspective, the approach tends to be that modest-performing GPs are visited more than once a year. Our findings show that among these groups statistically significant improvements occur in completeness of registration and reaching the target, and that the magnitude is more pronounced than that found in the whole population (Supplementary Table S3 for the 2017–2018 period). Yet, these improvements were equally visible, regardless of the number of visitations. Although the magnitude of the change seemed more pronounced in the group visited twice or more, the differences between visitation groups were not statistically significant.

## Discussion

### Summary

In our observational study performed in primary care, we evaluated the value of practice visitations to integrated CVRM in primary care. We observed no improvement in practices that were visited two or more times compared with practices with no visitation or one visitation. However, in those practices performing according to the lowest 25% on registration and predefined targets, improvements were seen irrespective of the number of visitations. Ideally, one would like to investigate the possible association between improvement in registration and achieved outcomes and a reduction of cardiovascular events. Yet, the care programme and the care group's multidisciplinary registration were not a priori developed and designed for complete registration of clinical event outcomes.

### Strengths and limitations

This study has a number of strengths. First, the large number of participants leads to a high representativeness. Second, because data collection and visitations did not interfere with daily practice routine, this further adds to the generalisability. Third, we included only practices where the GP was employed during the study period, minimising bias owing to changes in practice organisation and caregiver.

The study has a number of limitations. First, it was a non-randomised, observational study leading to a substantial risk of confounding bias in the estimates of the effect of visitation. Yet, we did adjust for expected confounding factors in our analysis. Second, technical problems or lack of time may have contributed to the fact that targets might not have been registered but were still achieved, leading to an underestimation of the effect. Third, we realise that, as a consequence of the care group's decision to use binary cut-off points, reductions in SBP for example, remain unnoticed if the cut-off is not met, leading to underestimation of improvements in some patients. Fourth, prescriptions of blood pressure-lowering and lipid-modifying medication were used as performance indicators, without knowing whether medication was indicated, which could have led to some overestimation. Fifth, PoZoB supported affiliated practices for several years in various ways, which have been described in detail, resulting in annual improvements of performance.^9^ This might have contributed to the lack of effect on performance of additional practice visitations.

### Comparison with existing literature

The results of our study are in contrast with a systematic review in 2012 that concluded that A&F overall leads to positive but highly variable effects.^5^ A&F showed positive effects in some studies on hypertension management and prescription rate,^11–14^ while in other studies on adherence to guidelines and decrease in first-ever strokes it did not.^15,16^ Comparison of these studies with our study is, however, difficult because of the heterogenous designs, interventions tested, and the difference in primary outcome. There are a number of reasons that possibly contribute to the lack of improvements in our study. First, overall mean registration rate was already high in 2016,^17^ making it difficult for practices to realise further improvements. Second, absenteeism owing to illness of primary care staff and lack of replacement can ensure that regular follow-up is lagging behind, resulting in poorer registration.^18^ Third, owing to privacy restrictions, it was not possible to take caregivers' characteristics, such as age, sex, and number of years working as a GP, into account. Finally, GPs and PNs may have clinical considerations for not reaching targets such as older age combined with side effects of medication, polypharmacy, the patients’ reluctance to intensify therapy, and near-target indicators.^19^

### Implications for research and practice

Research showed that feedback was more effective if performance was low, if given face to face by a colleague, on more than one occasion, and with well-defined goals.^6,20^ The current study showed that feedback given during annual visitations was not of additional value regarding improvements in clinical performance. Practice visitations however, could be used (i) to explore whether the general practice is motivated to improve; (ii) to explore what a practice needs to improve; and (iii) to emphasise the non-punitive character of A&F in order to maintain a trustful relationship. Future research could focus on the reasons why GPs and PNs deviate from current guideline targets. Furthermore, individual patient targets registered as such in the care group's multidisciplinary registration system, should be taken into account when evaluating practice performance. Finally, given the fact that practice visitations are a time-consuming, and therefore costly, operation, care groups may consider other possibilities to support healthcare providers, such as interventions aimed at improving patient self-management skills, taking the patient's preferences into account, and considering the patient as a partner, as this patient-centred approach focuses less on performance but more on a better health-related quality of life and more satisfied healthcare providers.^21,22^

In conclusion, practice visitations in our setting did not seem to lead to improvements in practice performance, nor in completeness of registration of risk factors or in reaching predefined target goals for cardiovascular risk factors.

## Data availability

Data for this article were provided by the PoZoB primary care group. Data will be shared on request to the corresponding author with the care groups’ board permission.

## Data

Data for this article were provided by the PoZoB primary care group. Data will be shared on request to the corresponding author with the care group's board permission.
